# Allosteric Theory: Taking Therapeutic Advantage of the Malleable Nature of GPCRs

**DOI:** 10.2174/157015907781695973

**Published:** 2007-09

**Authors:** Terry Kenakin

**Affiliations:** Dept. of Biological Reagents and Assay Develpoment, GlaxoSmithKline Research and Development, 5 Moore Drive, Research Triangle Park, NC 27709, USA

## Abstract

The description of the allosteric modification of receptors to affect changes in their function requires a model that considers the effects of the modulator on both agonist affinity and efficacy. A model is presented which describes changes in affinity in terms of the constant α (ratio of affinity in the presence *vs* the absence of modulator) and also the constant ξ (ratio of intrinsic efficacy of the agonist in the presence *vs* absence of modulator). This allows independent effects of both affinity and efficacy and allows the modeling of any change in the dose-response curve to an agonist after treatment with modulator. Examples are given where this type of model can predict effects of modulators that reduce efficacy but actually increase affinity of agonist (i.e. ifenprodil) and also of modulators that block the action of some agonists (the CXCR4 agonist SDF-1α by the antagonist AMD3100) but not others for the same receptor (SDF-1α peptide fragments RSVM and ASLW).

‘All models are wrong…but some are useful…’

anonymous environmental scientist

## INTRODUCTION

Heptahelical (also referred to as seven transmembrane, 7TM, G-protein coupled) receptors are nature’s prototype allosteric protein. They are designed to bind neurotransmitters, hormones, autacoids and other signaling molecules in one loci and transmit the information contained therein to another loci where other cellular partners such as G-proteins or β-arrestin interact. This information is transmitted as a change in the tertiary shape of the receptor to alter the affinity of these cellular partners after ligand binding. In fact, the change in rate of binding of G-proteins has been directly measured using plasmon-waveguide resonance spectroscopy. Specifically, studies on the δ-opioid receptor show that agonists and inverse agonists produce 50-fold changes in affinity for the G-protein subunit Gα_0_ [1], all this from the binding of a molecule some distance away from the receptor/G-protein site of interaction. This paper will discuss the allosteric nature of these receptors, the mechanisms by which small molecules can influence large protein tertiary structure, and the models used to describe and predict these effects.

## SYSTEM CAPABILITY

The first question is to consider what heptahelical receptors are capable of as they interact with various components of the cell. One of the key factors to consider is that, like all proteins, receptors can adopt numerous tertiary conformations. Since they also interact with a number of other cellular proteins, they have the capability of functioning as sophisticated signaling processing units. The idea that receptors form numerous conformations according to thermal energy in the system comes from theoretical considerations of protein structure [22,23,16,17,56,57] and also direct experimental observation. For example, studies with the prototype protein structural model for receptors, rhodopsin, indicate the existence of multiple conformations when interacting with rhodopsin kinase and arrestin [38]. Similarly, fluorescence spectroscopy has furnished evidence that the β_2_-adrenoceptor forms distinct ligand-specific conformations [18,19,51] while binding and kinetic experiments support multiple receptor conformations as well [30,48,49]. 

Heptahelical receptors reside in cell membranes with a series of domains facing the extracellular and another series facing the intracellular space. Until the last decade, the primary focus for receptor signaling has been heterotrimeric G-proteins. In this regard, there is a rich array of signaling possibilities for pleiotropic receptors binding to numerous types of G-proteins to release alpha and beta-gamma subunits into the cytosol. However, in the last few years,there have been other receptor partners detected that can react to activated receptor and behave as signal sources for the cell. For example, β-arrestin, once thought to mediate only receptor internalization and uncoupling from G-proteins, is now known to act as scaffolding for extracellular signal related kinases (ERK’s) to function as a signaling system in its own right [33,34,53]. In addition, numerous other proteins are being discovered that either allow information to be transmitted into the cell through interaction with receptors or couple to receptor to modify the effects of agonists. Thus, binding domains on receptors other than those responsible for interaction with G-proteins (i.e. PDZ, SH2, SH3 domains) interact with various intracellular signaling molecules [5]. Thus the stage is set for fine chemical control of the complex heptahelical signaling systems for therapeutic advantage; the pharmacological question is, how can this be achieved?

## LIGAND CAPABILITY

A key to understanding how small druglike molecules can influence large receptor proteins is the concept of conformational selection [6]. This idea describes the binding of a ligand to a range of receptor conformations according to its respective affinities for each conformation; this stabilizes the bound conformation(s). If these conformations are interconvertible, then it will be thermodynamically favorable for the system to produce more of that conformation at the expense of others, in essence the ligand will cause enrichment of its favorite conformation (in accordance with Le Chatelier’s principle). If some of these conformations have biological activity, then the ligand will confer this biological activity upon the system containing the receptor. This can be illustrated with a simple example for a system containing two protein states (R and R* where the ratio [R*]/[R] is defined by the allosteric constant L):

(1)
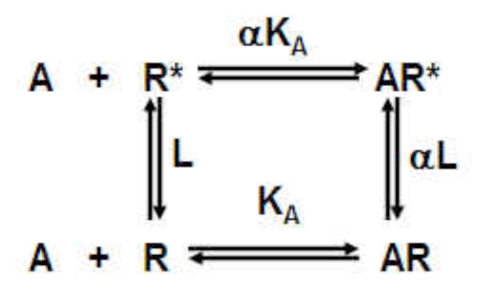

         

The ligand A binds to R with an affinity K_A_^-1^ and to R* with an affinity αK_A_^-1^. In the absence of ligand, the ratio of R* as a fraction of total receptor species ([R_total_]= [R] + [R*] + [AR] + [AR*])is given by ρ_0_ = L/(1+L) and in the presence of a saturating concentration of ligand ([A]  →∞) as ρ_∞_ =(α(1 + L))/(1 + αL). The effect of a ligand changing the ratio of R* to R is then given by:

(2) $$\frac{\rho_{\infty }}{\rho_{0}}=\frac{\alpha \left(1+L\right)}{\left(1+\alpha L\right)}$$

It can be seen from the condition α ≠ 1, (the ligand has differential affinity for the two conformations) that the ratio of the states necessarily will change upon ligand binding. The application of this concept to more complex systems of multiple conformations leads to a similar mechanism for ligand control. Assuming a system of conformations R and multiple other conformations labeled R_i_ where i=1 to n controlled by allosteric constants L_i_ to L_n_., the fraction of receptors not in the R state in the absence of ligand is given by:

(3) $$\rho_{\text{nonR}}=\frac{\sum_{i=1}^{n}L_{i}}{\left(1+\sum_{i=1}^{n}L_{i}\right)}$$

The fraction of receptors not in the R state in the presence of a saturating concentration of ligand (where the affinity for R is K^-1^) and for each state R_i_ as (Ψ_i_K ,)^-1^

(4) $$\rho_{\mathrm{nonR}}=\frac{\sum_{i=1}^{n}L_{i}+\left[A\right]/K\sum_{i=1}^{n}\Psi_{i}L_{i}}{\left[A\right]/K\left(1+\sum_{i=1}^{n}\Psi_{i}L_{i}\right)+\left(1+\sum_{i=1}^{n}L_{i}\right)}$$

This equation shows that no change in the fraction of receptors different from the R state will occur in the presence of the ligand only if the affinities of the ligand for every state is the same (equal to K, i.e. Ψ_ i to n_ = 1).In contrast, any differential affinity (Ψ_i to n_ ≠ 1) will result in a change in the ratio of conformational states, i.e. the ligand will alter the conformational makeup of the collection. This underscores the probability of changes in ensembles with increasing conformational states, i.e. the more states there are, the greater the probability that a ligand will induce a change. 

## MODELING ALLOSTERIC RECEPTOR EFFECTS

**Fig. (1):**
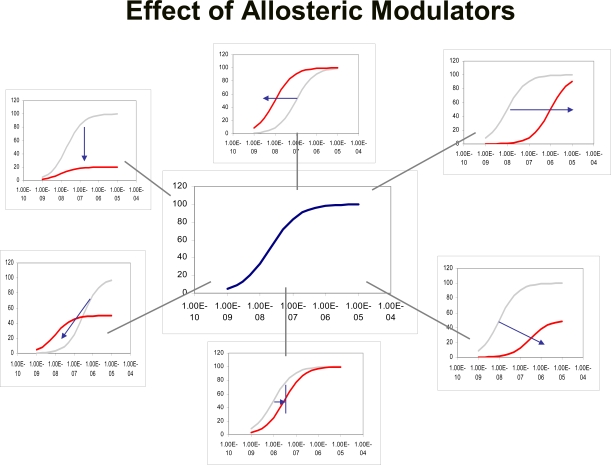
A sampling of the many possible effects allosteric modulators may have on the concentration-response curve to an agonist. Independent increases or decreases in the location parameter and maximal abscissae of these curves as well as incomplete saturable effects (limited shifts of the curve or changes in maxima) are possible. In effect, the allosteric modulator theoretically can create a completely new receptor with unique responsiveness to various probes such as agonists and/or radioligands.

There have been several models presented to account for and quantify allosteric effects on receptors. An allosteric change to a receptor essentially produces a new receptor which, in turn, may have completely different reactivities toward agonists and antagonists. Thus, a model must accommodate all changes to the original agonist concentration-response curve (see Fig. **1**). 

One of the earliest and most cited models for allosterism in receptors was presented by Ehlert [13]. It describes a receptor that can bind a probe molecule A and an allosteric modulator B with respective affinities K_A_^-1 ^and K_B_^-1^; the affinity of the each is modified by a factor α when the other is already bound to the receptor:

(5)
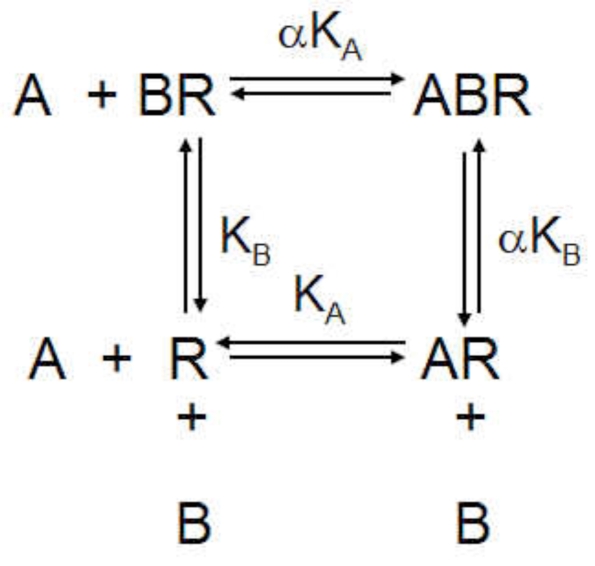

      

**Fig. (2):**
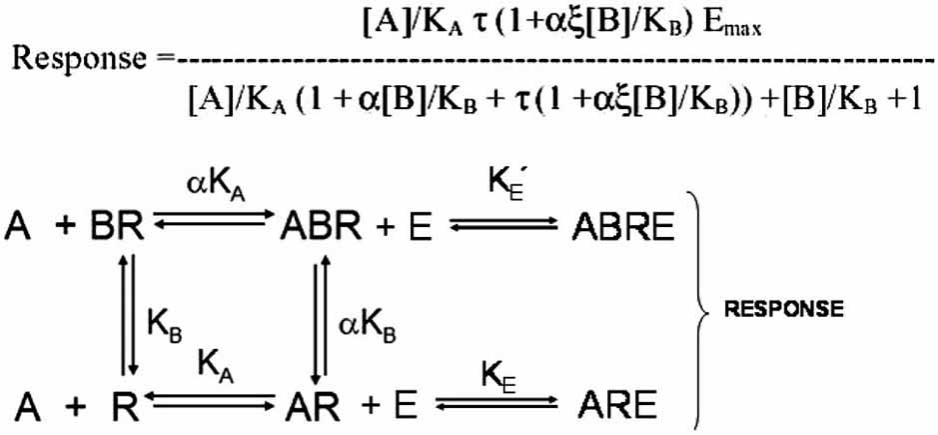
A simple theoretical model of allosteric receptor function whereby the receptor activates cellular response machinery according to an operational equilibrium dissociation constant K_E_ under normal circumstances and with a dissociation constant K^’^_E_ when bound to the allosteric modulator.

The allosteric effect is quantified by the cooperativity constant α which is the ratio of the affinity of the probe molecule for the molecule in the presence and absence of the modulator. For example, if α=10, then the affinity of the receptor for the probe is increased 10-fold when the modulator is bound. This model, when combined with the Operational model for receptor function [4], yields a useful model to describe allosteric functional effects (see Fig. **2**) [25,26]. The amalgam of the Ehlert model and the Operational model can produce virtually any change in an agonist concentration-response curve; the equation for agonist response with this model is given by:

(6) $$\text{Response}=\frac{\left[A\right]/K_{A}\tau \left(1+\alpha \xi \left[B\right]/K_{B}\right)E_{\text{max}}}{\left[A\right]/K_{A}\left(1+\alpha \left[B\right]/K_{B}+\tau \left(1+\alpha \xi \left[B\right]/K_{B}\right)\right)+\left[B\right]/K_{B}+1}$$

where α is the ratio of equilibrium dissociation constants of the agonist-receptor complexes (quantifying changes in agonist affinity) and ξ refers to the ratio of τ values (efficacy) of the agonist in the particular system in the presence and absence of modulator (ξ=τ’/τ where τ’ is the efficacy of the agonist in the presence of the modulator). Modulator-induced changes in either affinity or efficacy on concentration-response curves to agonists in systems of low sensitivity (low receptor density and/or low efficiency of receptor coupling) or high sensitivity are shown in Fig. (**3**). 

There is no reason to suppose that an allosteric modulator will only affect affinity or efficacy of the probe molecule but rather, a mixture of effects may result. Under these circumstances, the behavior of a concentration-response curve under the influence of a modulator may not indicate whether affinity, or efficacy (or both) are affected. For example, equation 6 predicts that the location parameter of the agonist concentration-response curve (EC_50_) depends both upon effects on affinity (α) and efficacy (ξ).

(7) $${\mathrm{Limit Relative K}}_{\text{obs}}=\frac{\left(\alpha +\tau \alpha \xi \right)}{\left(1+\tau \right)}$$

Therefore it will not be evident from shifts in the concentration-response curves whether the modulator affects affinity, efficacy or both. However, there are certain conditions and approaches which may allow isolation of certain effects. For example, in cases where partial agonism is observed it can be shown that changes in the maximal response to the agonist are dependent only upon modulator-induced changes in efficacy:

(8) $$\text{Limit Relative Max Resp}.=\frac{\xi \left(1+\tau \right)}{\left(1+\tau \xi \right)}$$

**Fig. (3):**
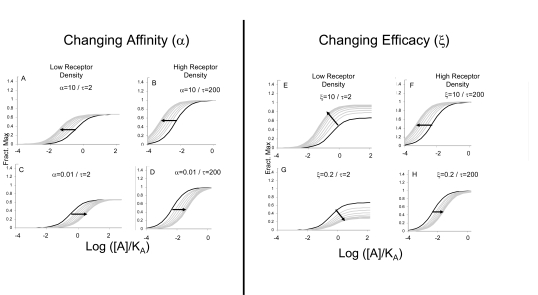
Effects of different modulators on agonist response according to the model depicted in Fig. (**2**). Changes in affinity (α) only produce changes in the location parameter of the concentration-response curves but no change in maxima. Modulator-induced changes in efficacy (ξ) can alter location parameters and maximal response. For allosteric potentiation of response, only changes in location are observed for full agonists For partial agonists, changes in maxima can be seen. In cases of allosteric reduction in efficacy, both changes in location and maxima may be observed.

It can also be seen that in cases where the agonist already is demonstrating full agonism (high τ), modulator-induced changes in efficacy may be masked (i.e. for high τ, Limit Relative Max Resp → 1; see Fig. **3F** and **H**). A key experimental strategy that can be effective is the use of high and low sensitivity assays to assess effects on agonists. Thus, low receptor density (or low efficiency receptor coupled system) may yield systems where the agonist is a partial agonist (i.e. produces sub-maximal system maximal response). Under these circumstances, pure effects on affinity can be separated from those on efficacy by the fact that an affinity effect will cause a dextral displacement of the partial agonist curve with no change in maxima (see Fig. **3A** and **C**). In contrast, effects on efficacy will result in a change in the maximal response of the partial agonist; some of these effects are shown in simulation in Fig. (**3E**) and (**G**). Similarly, high receptor density (or high efficiency receptor coupled system) yield full agonism for most agonists and can detect limiting levels of antagonism. As well as being a harbinger of allosteric effect in general, this can also reveal effects on efficacy. Specifically, non-zero values of ξ (efficacy is not zero when modulator bound) may be indicated when the modulator fails to completely suppress the agonist response (Fig. **3G**). 

**Fig. (4):**
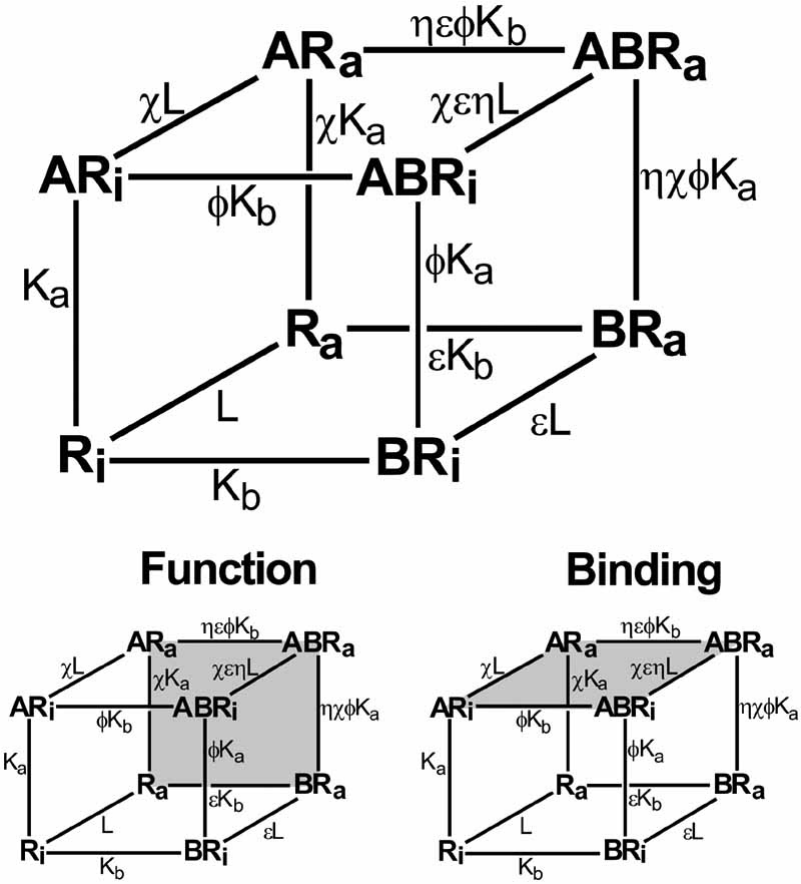
The Hall model [20] of allosteric function whereby the effect of the modulator on agonist-induced (A) or spontaneously formed active state receptor is affected by binding of the modulator (B). Shaded squares represent the respective species measured in functional and binding experiments. The fact that these species are different for the assay types leaves open the possibility that different responses to allosteric modulators may be observed in these two different assay formats.

Detailed model-driven analyses of allosteric effects have been presented elsewhere. An extensive experimental strategy to delineate effects on affinity and efficacy by allosteric modulators based on modeling is given by Ehlert [11] where a concise description of modulator effects can be obtained through the use of a ‘relative activity’ factor. Another model by Hall [20] gives a useful delineation of allosteric effects on receptor occupancy and activation. This model also outlines mechanistically how it may be possible to see dissociation of ligand function and binding. Specifically, an allosteric ligand may not affect the overall binding of a ligand (such as an agonist) but could block its’ function in terms of producing pharmacological response (no effect on α but ξ=0; see Fig. **4**). The receptor can exist in an inactive ([R_i_]) and activated ([R_a_]) form, both interacting with ligands [A] and [B]. If the radioligand is [A] and the allosteric modulator is [B], then a radioligand binding assay measures the fraction of total receptor bound to the ligand A (species [AR_i_], [AR_a_], [ABR_a_] and [ABR_i_]) according to the equation [20]:

(9) $$\rho_{\text{AB}}^{\text{*}}=\frac{\left[A^{\text{*}}\right]/K_{A}\left(1+\chi L+\phi \left[B\right]/K_{B}\left(1+\chi \eta \varepsilon L\right)\right)}{\left[A^{\text{*}}\right]/K_{A}\left(1+\chi L\left(1+\phi \eta \varepsilon \left[B\right]/K_{B}\right)+\phi \left[B\right]/K_{B}\right)+L\left(1+\varepsilon \left[B\right]/K_{B}\right)+\left[B\right]/K_{B}+1}$$

However, a functional assay measures the fraction of receptor species in the activated state ([R_a_], [AR_a_], [BR_a_] and [ABR_a_]). Under these circumstances, pharmacological response is given by [20]:

(10) $$\rho_{f}=\frac{\left[A^{\text{*}}\right]/K_{A}\left(\chi L\left(1+\phi \eta \varepsilon \left[B\right]/K_{B}\right)\right)+L\left(1+\varepsilon \left[B\right]/K_{B}\right)}{\left[A^{\text{*}}\right]/K_{A}\left(1+\chi L\left(1+\phi \eta \varepsilon \left[B\right]/K_{B}\right)+\phi \left[B\right]/K_{B}\right)+L\left(1+\varepsilon \left[B\right]/K_{B}\right)+\left[B\right]/K_{B}+1}$$

Measurement of the different receptor species by binding and functional assays accounts for differences that may be seen in binding and functional effects of allosteric modulators. Referring to Fig. (**4**), an allosteric antagonist with ε<1 and ф>1 can demonstrate dissociation between effects on binding and function by reducing the active state receptor (due to ε<1) but increasing the affinity of the antagonist-bound receptor for the agonist/radioligand (ф>1). Under these circumstances, functional response will decrease but binding of radioligand will be unchanged. 

A different kind of model has been used to predict receptor conformational states. Whereas the previous models are all linkage models where the receptor species are defined, this model does not define exact conformations but rather discusses probabilities of forming different states in response to ligand binding [39,40]. This ‘probabilistic model’ can be used to calculate the forces controlling the macro-affinity of a ligand for a collection of receptor conformations and also the effects of ligand binding to change the makeup of that collection of states (efficacy). This theory predicts that there is a positive correlation between ligand affinity and efficacy and, this was in fact observed in a simulation [28]. 

## THE IMPLICATIONS OF ALLOSTERISM

It can be seen from the foregoing discussion that an allosteric modulator can alter the affinity and efficacy of a probe molecule (agonist) for a receptor; as noted previously, the receptor essentially can become a different receptor with respect to that probe. This has implications for the detection and therapeutic use of allosteric modulators and also theoretically can give allosteric modulators the power to alter large protein-protein interactions. This is because the allosterically stabilized receptor may be different from the native receptor in a number of regions, not just the binding domain and this, in turn, may affect processes that utilize more than one region of interaction between the receptor and other proteins. For example, blockade of HIV infection is not amenable to single point mutation [12]. In fact, mutational studies implicate all four extracellular domains of CCR5 in the process of fusion of the virus with cell membranes [2, 3, 11, 32, 41, 43, 44]. The binding partner in HIV infection is a viral coat protein gp120 and point mutation studies on this protein also indicate that multiple regions of gp120 are involved with CCR5-mediated HIV infection [3, 31, 42, 43, 50]. In spite of the fact that huge protein-protein interactions mediate HIV entry, allosteric modulators such as aplaviroc [8,9] can block this multisite interaction with nanomolar potency by stabilizing an allosteric conformation of the CCR5 receptor [55]. This serves as a proof of concept that the allosteric stabilization of a receptor conformation can have powerful effects on the interactions of large proteins.

Orthosteric antagonists that block the probe binding site produce a uniformly unresponsive receptor, i.e. blockade of the receptor with any orthosteric antagonist results in an equally unresponsive receptor. Under these circumstances, the nature of the orthosteric antagonist is interchangeable, i.e. as long as the ratio of concentration to K_B_ (equilibrium dissociation constant of the antagonist-receptor complex) is the same, the pharmacological effect will be the same. This is not necessarily true of allosteric modulators. For example, aplaviroc, Sch-C and TAK779 are allosteric blockers of HIV entry. However, when probed with specific antibodies of CCR5, it can be seen that these HIV entry blockers produce differential binding profiles, i.e. specific probing of the receptor conformation reveals differences in conformation between the allosterically modified receptors [35]. Thus, with a range of allosteric modulators, the allosterically modified receptors may differ from each other but be otherwise similar with respect to a function such as HIV entry [27]. This may have implications for the development of resistance. It is predicted that mutation of HIV is capable of overcoming sensitivity to allosteric modification of receptors under selective pressure imposed by chronic use of an allosteric antagonist [10, 21, 36, 47]. For example, prolonged incubation *in vitro* with the HIV entry inhibitor AD101 leads to resistant strains of HIV-1. through an ‘escape mutant’ that is insensitive to blockade by this antagonist [54]. However, it would be predicted that these resistant strains will be sensitive to another allosteric modulator since a different conformation may be presented by a new allosteric ligand. In this way, the texture in antagonism imparted to a receptor by an allosteric modulator may offer an advantage over orthosteric blockers [27]. 

**Fig. (5):**
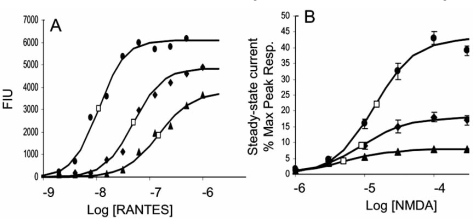
Effects of allosteric modulators on agonist response. A. Effect of Sch-C on CCR5-mediated calcium responses to the chemokine RANTES. Curves shown in the absence (filled circles) and presence of Sch-C (17.6 nM; filled diamonds : 26.3 nM filled triangles). Open squares indicate half maximal location parameters of the curves; note the dextral displacement with antagonsm by Sch-C. B. Blockade of NMDA responses of rat cortical neurons by Ifenprodil. Curves shown in the absence (filled circles) nnd presence of ifenprodil (0.1 μM, filled diamonds: 1 μM, filled triangles). Open squares indicate half maximal location parameters for the concentration-response curves; note the sinistral displacement with increasing concentrations of ifenprodil. Data redrawn from [29].

**Fig. (6):**
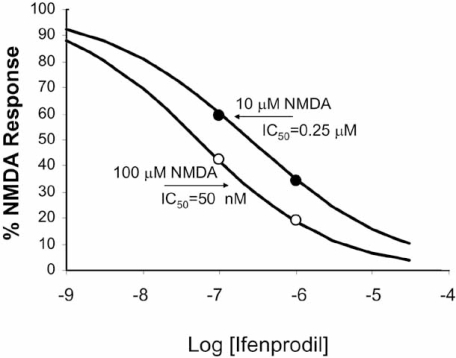
Inhibition of the effects of two concentrations of NMDA (see Fig. **5**) by ifenprodil. It can be seen that the IC_50_ of ifenprodil for NMDA inhibition actually decreases with increasing concentrations of NMDA (the potency of ifenprodil is greater blocking 100 µM NMDA as compared to 10 μM NMDA). Data redrawn from [29].

Because an allosteric modulator allows agonist binding to the receptor, there can be differences in the resulting effect and allosterically-induced texture in antagonism can lead to other interesting properties. For example, antagonism can be achieved by either blocking affinity (reduce α) but not efficacy (ξ≥1), reducing effacing (decreased ξ) but not affinity (α≥1), or both. The net result can be a complex reflection of activity of the modulator. For example,the canonical view of orthosteric antagonism, or antagonism in general (decreased α or ξ) is that the stronger the stimulus, the more antagonist is required for blockade. This can be shown by the effects of Sch-C on responses to RANTES (Fig. **5A**) which reduces RANTES efficacy (Watson *et al.*, 2005). However, an interesting effect is observed with antagonists that reduce the efficacy of agonists but concomitantly increase the affinity of the receptor for the agonist. Fig. (**5B**) shows the effects of ifenprodil on responses of rat cortical neurons to the agonist NMDA. In this case, it can be seen that while the maximal response is depressed, the EC_50_’s of the curves actually shift to the left indicating an increase in affinity of NMDA receptors for NMDA [29]. An examination of the schematic figure showing the reciprocal effects of modulators and probes on receptors (see schematic 5) indicates that, if ifenprodil increases the affinity of the receptor for NMDA (α > 1), then NMDA will necessarily increase the affinity of the receptor for ifenprodil. This means that the probe actually increases the activity of the modulator. This can be shown by the inhibition curve of ifenprodil calculated in the presence of two concentrations of NMDA (Fig. **6**). It can be seen that the modulator becomes more potent with increasing agonist concentration [29]. Thus, modulators have the ability to adjust their activity according to the magnitude of the signal input, a phenomenon referred to as ‘activation-dependent’ blockade.

This can be seen from an examination of equation 6 from which the following relationship can be derived:

(11) $$\frac{{\text{IC}}_{50}}{K_{B}}=\frac{\left(\left[A\right]/K_{A}+1\right)}{\left(\alpha \left[A\right]/K_{A}+1\right)}$$

Equation 11 predicts that if the modulator greatly reduces the affinity of the receptor for the probe (agonist) (α<<1), then the denominator → 1 and the expression reduces to the Cheng-Prusoff correction for simple competitive antagonism [7]. This means that the modulator will behave essentially as an orthosteric antagonist; whenever a modulator molecule is bound, the affinity of the receptor for the agonist is so low that essentially it is equivalent to an orthosteric occlusion of the agonist binding site. However, the interesting aspect of equation 11 is that, if the modulator ***increases*** the affinity of the agonist for the receptor (α>1), then the potency of the modulator as a blocker of effect will actually ***increase*** with increasing agonist concentration (as seen in Fig. **6**). 

It is useful to consider the differences between standard orthosteric antagonists (bind to the same site as the receptor probe) and allosteric modulators in terms of two important allosteric concepts, namely probe dependence and saturation of effect. Probe dependence refers to the fact that a conformational change produced by an allosteric modulator can have completely different effects on different receptor probes, i.e. a change in shape that is devastating to the activity of one probe may have no effect at all (or even produce potentiation) on another. For example, the muscarinic receptor allostesric modulator eburnamonine produces a 30-fold potentiation of responses to pilocarpine (EC_50_ for pilocarpine is decreased 30x), no change at all to the activity of arecaidine propyl ester and a 15-fold blockade of the agonist arecoline (EC_50_ arecoline is decreased by a factor of 15) [24]. Saturation of effect reflects the fact that, unlike a process such as competition for a common binding site, the binding of allosteric modulators to their own site on the receptor means that the allosteric effect reaches a maximal asymptote upon saturation of the allosteric binding site. Therefore, the allosteric effect is finite with a magnitude determined by (in terms of the functional Operational allosteric model) the magnitude(s) of α and ξ. It is worth examining the practical consequences of these properties.

## PROBE DEPENDENCE

The major implication of this effect is that the physiologically relevant probe molecule should be used for all characterizations of an allosteric molecule for test data to be predictive therapeutically. Effects on a stable laboratory test probe (chosen for practical experimental reasons) may not be predictive of the natural ligand. This idea extends to screening for new allosteric modulators. For example, the HIV entry blocker aplaviroc blocks the binding of the chemokine ligand ^125^I-MIP-1α but does not affect receptor occupancy by the chemokine ^125^I-RANTES [35,55]. Therefore, a radioligand displacement screen with ^125^I-RANTES would not have detected this molecule. 

Another implication of probe dependence is the potential for target salvage in cases where a biological target mediates a pathological function and physiologically important and useful functions, blockade of that target is contraindicated. However, an allosteric modulator has the potential to block the pathological input and leave the normal physiological function intact. In cases such as the use of the chemokine receptor CXCR4 for HIV entry in late stage AIDS [15], this may be important. There are knockout data to suggest that this receptor is functionally important, especially in early development [37,52,58]. Therefore, an allosteric modulator that blocks the usage of CXCR4 by HIV but leaves the receptor free to respond to its natural agonist Stromal Derived Factor 1α (SDF-1α) would be an advantage. There are intriguing data to show that while antagonists of CXCR4 such as the antibody T140 and receptor antagonist AMD3100 both block the chemokine CXCR4 receptor mediated signals of the natural agonist SDF-1α, they do not block the CXCR4-mediated signals of the peptide fragments RSVM and ASLW [46]. 

The principle of permissive antagonism is illustrated by the HIV blocking properties of aplaviroc. In this case, aplaviroc blocks HIV entry but allows the chemokine RANTES to bind unimpeded to the receptor. However, this salvage is incomplete in that RANTES function is blocked (α≈ 0.7, ξ=0 equation 6) [55]. In general, allosteric modulators give the potential of attacking all biological targets that cannot be prosecuted with standard orthosteric molecules. 

Another important principle associated with allosteric modulators is that of saturation of effect. Fig. (**7**) shows the inhibition of the chemokine ^125^I-MIP-1α binding .with non-radioactive MIP-1α and the small molecule ICB35625. It can be seen that while non-radioactive MIP-1α reduces the binding of the radioligand to basal levels, UCB35625 only produces a 30% reduction in binding [45]. This is a classic allosteric effect that reflects a slight reduction in affinity produced by the allosteric modulator. Effectively, such antagonists will reduce but not completely block physiological response, a unique effect that may be therapeutically useful in certain pathological conditions. 

## CONCLUSIONS

It can be seen that the promiscuous nature of 7 transmembrane receptors, with respect to coupling proteins in the cell membrane and in association with their allosteric nature, allows them to form different conformations, and this can produce a potentially sophisticated signaling system. The challenge now is to harness this sophistication through selective ligands to yield therapeutically useful profiles of drug activity. The key to this process is to have the eyes to see specific receptor behaviors, i.e. to have a range of assays capable of observing different aspects of receptor activity. In addition, theoretical models that describe and predict these effects are useful as indicators of the implications of the effects observed experimentally.

## Figures and Tables

**Fig. (7):**
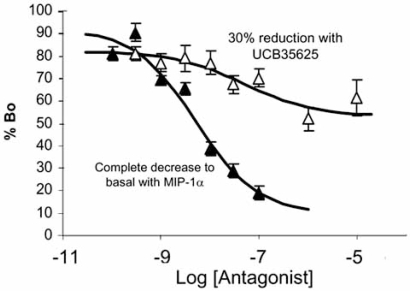
Displacement of bound ^125^I-MIP-1α from chemokine C receptors type 1 (CCR1) by MIP-1α (filled triangles) and the allosteric ligand UCB35625 (open triangles). Incomplete displacement indicates an allosteric resetting of the affinity of the receptor for MIP-1α. Redrawn from [45].
